# Case Report: Spontaneous Rupture of Hepatic Hemangioma

**DOI:** 10.3389/fmed.2022.918748

**Published:** 2022-07-13

**Authors:** Bing Pan, Shao-Cheng Lyu, Qiang He

**Affiliations:** Department of Hepatobiliary Surgery, Beijing Chaoyang Hospital, Capital Medical University, Beijing, China

**Keywords:** hepatic hemangioma, spontaneous rupture, surgery, emergency, intra-abdominal hemorrhage

## Abstract

**Background:**

Hepatic hemangioma (HH) is a congenital vasal malformation that seemed like the most probable benign liver neoplasm, composed of masses of blood vessels, which are anomalous in arrangement and size. In most cases, HH is asymptomatic, and patients have an excellent prognosis. According to research, the location and size of the mass are correlated with the symptoms and complications. Reports of spontaneous rupture of HH have been less reported in the literature. In this emergency condition, dynamic contrast-enhanced CT scanning, especially triple-phase computed tomography (CT) with delayed imaging, is preferred.

**Case Presentation:**

Here, we presented two middle-aged female patients with spontaneous rupture of HH in our hospital. Following an accurate diagnosis of enhanced CT and emergency surgery, patients recovered well and were discharged from the hospital.

**Conclusion:**

Appropriate imaging studies, especially enhanced CT, and emergency surgery are indispensable for patients with spontaneous rupture of HH. As a surgeon, we need to pay attention to the asymptomatic patient with HH.

## Introduction

Hepatic hemangioma (HH) is the most probable benign tumor of the liver, and often asymptomatic, can be found accidentally during the imagological examination, has a prevalence of approximately 20%, and is more frequent in women. Clinically, the most common type is a cavernous hemangioma, and most patients have an excellent prognosis because of the benign nature of hemangioma (1). So, most scholars propose that surgery should be restricted to the specific situation. The incidence of HH with rupture is low (1–4%). However, the mortality is rather high. Thus, spontaneous rupture is the most severe complication (2). There is no scientific evidence correlating the size of the hemangioma with the risk of rupture. In this emergency condition, dynamic contrast-enhanced computed tomography (CT) scanning, especially triple-phase CT with delayed imaging, is preferred. Herein, we presented two patients in our hospital, whose HHs were spontaneously ruptured, and whose hemangiomas are less than 10 cm in diameter. Following an accurate diagnosis of enhanced CT and emergency surgery, patients recovered well and were discharged from the hospital.

## Case Presentation

### Case 1

**FIGURE 1:**
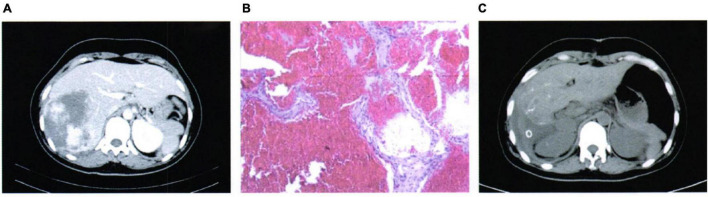
Case 1: **(A)** Pre-operative imaging examination, **(B)** pathological findings, and **(C)** post-operative imaging review.

A 33-years-old woman was admitted to the emergency department with persistent hurt in the upper quadrant of the abdomen, which had occurred suddenly, and the pain was not relieved after resting or changing position. She denied a history of any medical condition (including hepatitis), recent trauma, or oral contraceptive use, and a family history of hepatic disease. On admission physical examination (PE), the following results are observed: temperature (T) is 36.5°C, heart rate (HR) 75 beats/min, respiratory rate (RR) is 20 times/min, and blood pressure (BP) is 110/75 mmHg. Tenderness in the right upper quadrant of the abdomen was noted. Cardiopulmonary function examination showed no obvious abnormalities. Laboratory examination results showed a hemoglobin (Hb) level of 98 g/L (normal range: 120–160 g/L). The rest of the laboratory tests, including liver function and coagulation function, are normal. The abdominal enhanced-CT result (Figure 1A) showed a large round shape with a slightly lower confounding density shadow in the right hepatic. During the venous phase and the delayed phase, the enhancement is obvious, showing a tendency for inward filling, the local capsule is blurred, and a small amount of effusion is seen around the liver. According to the imaging study, we considered a diagnosis of rupture and hemorrhage of HH. Considering the threat of further rupture of the hemangioma, the patient underwent an emergency exploratory operation. During the operation, a dark red mass of about 10 cm was seen in the right liver, which was tough in quality. The mass slightly adheres to the diaphragm and lateral abdominal wall, and a small amount of hemorrhage can be seen around the liver. So, HH resection was performed. The findings of pathology indicated rupture hemorrhage of HH (Figure 1B). The patient recovered well (Figure 1C) and was discharged 5 days post-surgery.

### Case 2

**FIGURE 2:**
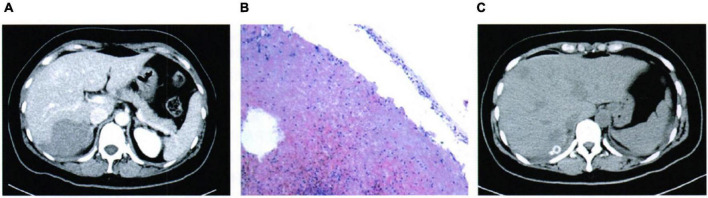
Case 2: **(A)** Pre-operative imaging examination, **(B)** pathological findings, and **(C)** post-operative imaging review.

A 36-year-old woman was admitted to the clinic with a chief complaint of “sudden right the upper quadrant pain for 14 h.” PE indicated abdominal pain, especially in the upper right abdomen. There was no obvious muscle tension and rebound pain. The results of the laboratory were normal. The enhanced abdominal CT said a mass in the right liver lobe, which was a patchy progressive enhancement shadow, a fusiform shape was visible in the adjacent liver capsule, and there was a small amount of fluid around the liver (Figure 2A). The patient underwent exploratory laparotomy followed by liver segmentectomy. The pathology results revealed rupture hemorrhage of HH (Figure 2B). The post-operative outcome was favorable (Figure 2C).

## Discussion

Hepatic hemangioma is the most probable benign neoplasm and is often found in women. The majority of HH is a cavernous hemangioma, with little chance of malignant transformation (3). After improving people’s health consciousness, more and more patients with hemangioma have been found. HHs grow slowly, and the course often lasts for many years. In most cases, the diameter of HH (<4 cm) is usually asymptomatic and easy to ignore, being discovered only as an incidental imaging finding. At >4-cm diameter, HHs are considered gigantic in size and might result in symptoms, such as abdominal discomfort, rupture, internal hemorrhage, coagulation disorder, etc. (4).

Reports of HH with spontaneous hemorrhage are less, causing a misunderstanding and ignorance of its serious complications (5). The incidence of abdominal bleeding caused by ruptured HH is low (1–4%), the mortality ranges from 60 to 70% (2), and the surgery mortality rate from this complication is 36.4% (6). There has been no scientific evidence correlating the size of the hemangioma with the risk of rupture. The first spontaneous rupture case of HH was reported by Van Haefen in 1898 (7). Yamamoto et al. documented 28 cases of ruptured tumors, with sizes ranging from 3 to 25 cm (8). To more comprehensively understand this disease, we searched PubMed and found 13 cases reported in recent 20 years (Table 1). These previous reports, combined with our case report, will significantly contribute to the diagnosis and treatment of spontaneous rupture of HH.

**TABLE 1:** Other case reports of spontaneous rupture of hepatic hemangioma (HH) reported in PubMed in recent 20 years.

Age	Gender	Size (cm)	Hemorrhage type	Management	outcome	Author	Title	Journal	year
37	Female	15×12	Intratumor bleed	Hepatectomy	Survival	Zhao (12)	Spontaneous rupture of hepatic hemangioma: a case report and literature review	Int J Clin Exp Pathol	2015
56	Female	10×6	Extratumor bleed	Hepatectomy	Survival	Zhai (13)	Spontaneous rupture of giant hepatic hemangioma: misdiagnosis as gastrointestinal perforation	J Int Med Res	2019
35	Female	12×10	Extratumor bleed	TACE	Survival	Cao (14)	A case of spontaneous hepatic hemangioma rupture: Successful management with transarterial chemoembolization alone	J Interv Med	2019
65	Male	11×8	Intratumor bleed	Hepatectomy	Survival	Yang (15)	Spontaneous intracapsular hemorrhage of a giant hepatic cavernous hemangioma: a rare case report and literature review	BMC Gastroenterol	2021
25	Male	12×9	Extratumor bleed	Hepatectomy	Survival	Gupta (16)	Spontaneous rupture of a giant hepatic hemangioma-report of a case	Indian J Surg	2012
76	Female	9×9	Extratumor bleed	Hepatectomy	Survival	Nguyen (17)	Giant hepatic hemangioma rupture in a patient on direct oral anticoagulant therapy	J Surg Case Rep	2021
46	Female	*	*	Untreated	Dead	Bel Hadj et al. (5)	Spontaneous rupture of a hepatic cavernous hemangioma: A rare case of sudden unexpected death	Am J Forensic Med pathol	2020
64	Male	5	Extratumor bleed	TACE	Survival	Rossi (18)	Spontaneous hepatic hemangioma rupture and hemoperitoneum: a double problem with a single stage interventional radiology solution	Clin Exp Emerg Med	2019
52	Female	16	Intratumor bleed	Hepatectomy	Survival	Hao (19)	Spontaneous internal hemorrhage of a giant hepatic hemangioma: A case report	Medicine (Baltimore)	2017
31	Male	9.7×7.3	Extratumor bleed	TACE	Survival	Jain et al. (2)	Spontaneous rupture of a giant hepatic hemangioma–sequential management with transcatheter arterial embolization and resection	Saudi J Gastroenterol	2010
25	Female	*	Extratumor bleed	Hepatectomy	Survival	Santos Rodrigues (20)	Spontaneous rupture of giant hepatic hemangioma: a rare source of hemoperitoneum. Case report	G Chir	2010
54	Female	4.4×2.8	Intratumor bleed	Hepatectomy	Survival	Kim (21)	Hemorrhagic hemangioma in the liver: A case report	World J Gastroenterol	2015
70	Male	*	Extratumor bleed	Peritoneal drainage	Survival	Goidescu (22)	Ruptured liver cavernous hemangioma-rare cause of hemoperitoneum	J Med Life	2015

*Represent that there is no detailed description in the original text.

Clinical manifestation of spontaneous rupture of HH consists of sudden abdominal pain, anemia that is secondary to a hemoperitoneum, and disseminated intravascular coagulopathy (DIC). Spontaneous rupture of HH is considered a life-threatening condition (9), as its clinical signs are not usually specific. Dynamic contrast-enhanced CT scanning, especially triple-phase CT with delayed imaging, is preferred. Conservative treatment may result in hypovolemic shock, and emergent hepatic resection should be applied, although high operative mortality (10). Thus, emergency surgery is needed for patients with clinical symptoms, and high-risk complications should be more actively treated.

## Conclusion

At present, due to a poor understanding of the natural history of asymptomatic hemangiomas (11), patients might have died unexpectedly when hemangioma ruptured into the abdominal cavity without surgical treatment. An accurate diagnosis of a hemangioma as the cause of a hemoperitoneum would result in correct clinical decision-making and treatment. Thus, enhanced CT and emergency surgery are necessary for ruptured or bleeding HHs. We hope that our cases will attract more attention to this complication in clinical work. It requires us to reconsider indications for surgery.

## Data Availability Statement

The raw data supporting the conclusions of this article will be made available by the authors, without undue reservation.

## Ethics Statement

Written informed consent was obtained from the patient for the publication of this case report. Written informed consent was obtained from the patient for the publication of any potentially identifiable images or data included in this article.

## Author Contributions

BP and S-CL contributed to the planning and organization, collected clinical data, supervised the findings of this work, analyzed the results, and prepared the manuscript. QH aided in the data collection and supervision. All authors contributed to the article and approved the submitted version.

## Conflict of Interest

The authors declare that the research was conducted in the absence of any commercial or financial relationships that could be construed as a potential conflict of interest.

## Publisher’s Note

All claims expressed in this article are solely those of the authors and do not necessarily represent those of their affiliated organizations, or those of the publisher, the editors and the reviewers. Any product that may be evaluated in this article, or claim that may be made by its manufacturer, is not guaranteed or endorsed by the publisher.
